# A qualitative analysis of junior doctors’ journeys to preparedness in acute care

**DOI:** 10.1186/s12909-020-1929-8

**Published:** 2020-01-13

**Authors:** Samuel Burridge, Thurkaa Shanmugalingam, Fatima Nawrozzadeh, Kathleen Leedham-Green, Amar Sharif

**Affiliations:** 1 London North West University Healthcare NHS Trust, Northwick Park Hospital, Watford Road, Harrow, HA1 3UJ UK; 2 Imperial College London, Medical Education Research Unit, Sir Alexander Fleming Building, South Kensington Campus, Exhibition Road, London, SW7 2AZ UK

**Keywords:** Preparedness for practice, Acute care, Shadowing

## Abstract

**Background:**

New doctors are expected to assess and manage acutely deteriorating patients from their first days in the hospital. However, current evidence suggests that medical graduates are not prepared for this. We aimed to explore junior doctors’ first experiences with unwell patients and how they developed preparedness over time.

**Methods:**

We conducted seven semi-structured interviews with doctors in their first postgraduate year. The interview transcripts underwent inductive thematic analysis using consensual qualitative research approaches. Themes identified were categorised into early experiences of unpreparedness, first experiences of genuine preparedness, and making sense of how they became prepared. Reflection on how participants progressed between the two was facilitated through a sorting and ranking exercise.

**Results:**

Most participants initially felt unprepared when responding to acutely unwell patients. They described feeling overwhelmed, apprehensive and challenged. Two main challenges involved knowing when to escalate, and feeling expected to perform beyond their level of competency. A lack of acute care exposure at medical school was a common thread. All participants felt prepared to respond to unwell patients three to six months after starting work. Hands-on experience, reflection, simulation and multidisciplinary team-working were consistently ranked as the most useful learning experiences.

**Conclusion:**

Starting work as a doctor is a challenging time and preparedness to manage an acutely deteriorating patient is a common area of concern. As preparedness in acute care ranks poorly compared to other outcomes, we see this as an important area for improvement. Our findings suggest that undergraduates may lack sufficient opportunities for scaffolded decision making in acute care, and that increasing the intensity of clinical shadowing may improve preparedness and should inform future educational interventions.

## Introduction

New medical graduates in the United Kingdom, also known as foundation doctors, are expected to carry out a range of duties from their first day of work. This includes assessing and managing acutely unwell ward patients with time-critical conditions. Despite being specified in the General Medical Council’s (GMC) ‘Outcomes for Graduates’ [1], current evidence suggests foundation doctors are unprepared for the early management of patients with acute conditions [2]. It is an area in which foundation doctors perceive themselves to be the least prepared, with this perception shared amongst senior clinicians [3].

Foundation doctors have difficulties with tasks such as gathering relevant information, prioritising activities, knowing when to escalate and managing their emotional responses [2]. Inexperience of on-call duties as a medical student is associated with strong feelings of unpreparedness [2]. Furthermore, at the end of foundation year one, being the first doctor to respond to a deteriorating patient remains a significant area of concern [4].

Jen et al. [5] observed a 6% increase in mortality when comparing patients admitted on the same day that foundation doctors start work with admissions from the previous week. Team change-over is a complex time with loss of team structures and local tacit knowledge. However, junior doctor preparedness in acute care may be a contributing factor.

The literature to date has been mostly observational, identifying that graduates feel unprepared in acute care [2–4]. There is a gap in the literature for an explorative paper examining foundation doctors’ perceptions of preparedness after starting work and how these develop over time; as well as identifying and explaining which educational experiences they found more and less useful in increasing preparedness.

## Methods

### Setting and participants

We invited all foundation year one (FY1) doctors from a teaching hospital in London to participate. This hospital employs a total of 40 FY1 doctors, the majority of which trained at medical schools in the United Kingdom. FY1 doctors work across a range of medical and surgical specialities in 4 month attachments during their first year. Invitations were sent by email and announced at weekly junior doctor teaching sessions from December 2018 to February 2019.

### Data collection

An interview topic guide (Table 1) and sorting and ranking activity were developed collaboratively between the authors and clinical teaching fellow network of Imperial College London (18 active fellows in 2018–19). The interviews were semi-structured to openly explore participants’ experiences of acute care, what they conceived preparedness to be, and how they valued different learning experiences. The sorting and ranking activity involved a set of laminated cards, each labelled with a commonly used educational experience aimed at supporting preparedness in acute care (Table 2). As some novel or niche experiences may have been excluded, participants had a blank card which they could write on and add to the set. The interviews took place between March and June 2019 and were conducted by SB, FN and TS, all clinical teaching fellows with between three and four years’ postgraduate experience. Interviewers were not involved in foundation doctors’ assessment or appraisal and were trained by an experienced researcher (KL) to present and respond to topics in a neutral way, but to organically explore emergent areas of interest. Towards the end of the interview, participants were invited to sort the learning experience cards into one of four groups: essential, nice to have but not essential, not useful, or no experience of. They were then asked to rank and explain their essential experiences in order of importance.

**Table 1:** Interview topic guide

1. Thinking back to starting work as a junior doctor, can you tell me about the first time you had to respond to a deteriorating patient on the ward? 2. How did you feel when you were responding to the patient? 3. What did you find particularly challenging about the situation? 4. Overall, did you feel prepared? Why or why not? 5. What do you think has been most helpful in preparing you to respond to deteriorating patients? 6. In order to help feel more prepared in responding to deteriorating patients, are there any learning experiences you wish you had or had more of during medical school?

**Table 2:** Sorting and ranking activity

Educational experience	Frequency of selection as ‘essential’ by the 7 participants (frequency of ranking as first in essential column)
Formal undergraduate teaching	3 (0)
Hands-on experience	7 (5)
Informal peer-to-peer learning	1 (0)
MDT team-working	5 (1)
Mentorship	3 (1)
Self-directed study	3 (0)
Self-reflection on previous experiences	6 (0)
Simulation	5 (0)
Weekly postgraduate teaching	2 (0)
Workplace based assessments	2 (0)

### Data analysis

The interviews were audio-recorded, professionally transcribed and checked for quality by SB. All transcripts were anonymised before analysis. Data analysis was supported by Dedoose online software. A consensual qualitative research (CQR) approach was used [6] which combines elements from phenomenological [7], grounded theory [8], and comprehensive process analysis [9]. It is “an inductive method that is characterized by open-ended interview questions, small samples, a reliance on words over numbers, the importance of context, an integration of multiple viewpoints, and consensus of the research team. It is especially well-suited to research that requires rich descriptions of inner experiences, attitudes, and convictions” [6].

The first interview underwent open line-by-line coding jointly by SB, FN, TS and KL. The subsequent six interviews were open coded individually by SB, FN and TS, with each interview then cross-checked by a different author. Initial codes were then focused into themes using an iterative consensual process involving axial coding with constant comparison [10]. Emerging themes were discussed by SB, FN, TS and KL with differences in interpretation debated until a consensus was reached. Finally, the themes was categorised into early experiences of unpreparedness, first experiences of genuine preparedness, and making sense of how they became prepared.

### Ethical approval

Ethical approval was obtained from the Research and Development Office, London North West University Healthcare NHS Trust. The study objectives were explained to the participants and written consent was obtained before each interview.

## Results

Seven FY1s responded to the invitation and were interviewed (5 male and 2 female, aged 23–26 years). The interviews took place between seven to nine months from the FY1s’ start of work and lasted an average of 29 min. All major themes are illustrated with quotes; minor themes are listed and quotes only included if there was consensus that they were interesting or insightful.

### Early experiences of unpreparedness

Most participants initially felt unprepared in responding to deteriorating patients. They frequently described feeling ‘overwhelmed’, ‘apprehensive’, ‘unsettled’ and ‘out of [their] depth’. Participants did not feel confident in recognising what situations they would be expected to manage on their own as an FY1. Knowing when to escalate, and who to escalate to, were areas of significant concern. In parallel to this, participants felt that at times they were expected to perform beyond their level of competency. They also found situations more challenging when patients did not improve after initial management steps were started (Table 3).

**Table 3:** Early experiences of unpreparedness

Categories	Themes	Number of codes (number of respondents)	Example quotes
Emotions	Overwhelmed	10 (5)	*“Overwhelmed by the fact that there is a deteriorating patient and you are the main doctor.” (Respondent D)*
	Out of my depth	8 (3)	*“In F1, so I was apprehensive and then felt worried during the on-calls and also kind of out of my depth in dealing with the things that I was presented with.” (Respondent E)*
	Challenged	6 (4)	*“It was really hard, and it was really horrible for like, being a doctor for four days and then something like that to happen.” (Respondent B)*
	Apprehensive	5 (3)	*“Whereas it’s like now go and fend for yourself type thing. I think I was quite apprehensive, apprehensive probably, and unsettled by it as an experience.” (Respondent C)*
	Unsettled	6 (3)	*“I think in the first few on-calls I found them really upsetting like how unprepared I had felt for them.” (Respondent E)*
	Minor themes: Disassociation Adrenaline rush		*“Well I think sometimes you are so busy that you don’t kind of process how you are feeling.” (Respondent C)*
Main challenges they faced	When to escalate	9 (4)	*“I think probably most importantly is knowing when to escalate... I think but there’s a balance between okay, I can do the A to E and I can get all the data that I can think of and at what point does my management stop and there is nothing else that I am comfortable administering on my own? [At] what point do I need senior support?” (Respondent D)*
	Expectation to perform beyond level of competency	11 (4)	*“So, you were kind of forced to grow faster in your role and to be treated more or less like a heavily supervised SHO.” (Respondent C)*
	No improvement following initial management	3 (3)	*“I had never been in an experience to say, to see a patient go steadily downhill slowly over the course of the day and have outreach and ITU involved in his care. That’s, it’s usually just you know the unwell patient, acutely unwell patient, manage them, they get better.” (Respondent F)*
	Minor themes: Unsure on pace of management Difficulty managing families in an emergency		*“That was probably when it was quite emotional, his family were obviously distraught, they had very little faith in us…” (Respondent G)*
Making sense of why they were unprepared	Lack of exposure at medical school	8 (5)	*“So, that was the first time I saw something that was quite outside of what I had been used to seeing before I came into F1.” (Respondent F)*
	Unaware of diagnostic possibilities	7 (6)	*“I just didn’t know at that time what the possibilities were for what was causing that.” (Respondent E)*
	Managing failure of senior support	4 (2)	*“I eventually got through to the med reg, who was downstairs; it was quite a busy night for clerking... So, eventually, after a little while, he came up, reviewed the patient.” (Respondent G)*
	Little experience of prioritisation	4 (3)	*“So, it was quite a straightforward septic patient, complicated by the fact that I got two further bleeps; one about a potential MI and one about a potential re-bleed.” (Respondent D)*
	Preparedness is dependent on clinical scenario	11 (5)	*“I felt more prepared for things like sepsis or a drop in blood pressure... But the things I was called about like on the stroke ward like drop in GCS I felt really unprepared for because I just didn’t know at that time what the possibilities were for what was causing that, what I needed to do and how urgently.” (Respondent E)*
	Comfortable with ABCDE assessment	9 (3)	*“I suppose, actually kind of being grounded and thinking “well, I do know what to do, it’s just an A to E assessment, let’s start with that and let’s see where that takes us”.” (Respondent D)*
	Minor themes: Difficulty translating theory into practise More difficult making decisions in complex cases		*“…and knowing what kind of, how fast things need to happen, how much I needed to do, what I should do and what I should do if the person I was escalating to didn’t want to come were the challenging things.” (Respondent E)*

“Knowing where I’m out of my depth or knowing where I should be out of my depth and where I should just be improving myself… What is expected of me as an F1?” (Respondent G)

In making sense of why participants felt unprepared, we identified a number of contributing factors. A lack of exposure to acute care scenarios at medical school was a recurring theme. Participants felt they had little experience of prioritisation when responding to multiple unwell patients. Initial feelings of preparedness were dependent on the type of emergency situation; for example, participants felt more prepared for managing sepsis or hypotension and less so for reduced level of consciousness. Similarly, many participants admitted they were unaware of the diagnostic possibilities for certain problems. Participants also felt unsure of the timing that investigations or referrals should take place in an acute situation. However, participants did feel confident in using the ABCDE algorithm to structure their initial assessment and management.

“...just [in] real life what you should do and who you should call and how quickly, I had no idea.” (Respondent E)

### First experiences of genuine preparedness

All participants reported feeling genuinely prepared to respond to an acutely unwell patient after working for between three to six months. To them, feeling prepared was a combination of understanding the hospital, being comfortable with initial management steps, knowing when to escalate and recognising the seriousness of a situation (Table 4).

**Table 4:** First experiences of genuine preparedness

Categories	Themes	Number of codes (number of respondents)	Example quotes
What preparedness feels like	Confidence	10 (6)	*“And relax into it as it were… It’s not so much your mind saying, “oh god, I’m in this situation”, it’s your mind is now used to that situation.” (Respondent C)*
	Knowing the hospital environment and MDT	6 (4)	*“So yeah I just felt more part of the team, I’d had more of chance to build a rapport and understand the workings of a multidisciplinary team in this setting and this particular ward and department.” (Respondent A)*
	Knowing the initial management steps	5 (4)	*“I knew how to initiate management, I felt much more comfortable securing his airway, I was much happier delegating to nurses.” (Respondent B)*
	Knowing when to escalate	13 (6)	*“That was something I learned over time and now, I feel like I know, “Well, this is something that needs to have a MET call put out, this is something that needs to have a crash call put out… And I don’t feel like I was fully prepared for that when I first started working.” (Respondent G)*
	Recognising the seriousness of a situation	4 (2)	*“I feel much more prepared now, I feel like, I feel more able to recognise how serious a situation is and therefore not just panic at everyone.” (Respondent E)*

“I think probably most importantly is knowing when to escalate... I think but there’s a balance between okay, I can do the A to E and I can get all the data that I can think of and at what point does my management stop and there is nothing else that I am comfortable administering on my own? [At] what point do I need senior support?” (Respondent D)

Understanding the hospital incorporated both a familiarity of the hospital environment and processes as well as an appreciation of the multidisciplinary team (MDT) in acute situations.

“I always have that support of nurses and physios and everyone, and I think it just wouldn’t be the same if you didn’t take it on board. The nurses always know when something is going wrong and if you don’t listen to them then, yeah, it’s your own head on the line really.” (Respondent B)

### Making sense of how they became prepared

In developing preparedness in acute care, participants drew on a range of learning experiences (Table 5). Hands-on experience consistently ranked as the most essential, with reflection, simulation and MDT team-working also frequently ranked as essential learning experiences (Table 2). Participants felt they had little experience of decision making or taking ownership of an acute situation at medical school, and only developed this after beginning work.

**Table 5:** Making sense of how they became prepared

Categories	Themes	Number of codes (number of respondents)	Example quotes
Why experiential learning matters	Volume is essential	19 (7)	*“What is expected of me as an F1? I feel like I’ve got that down for sure, but it required multiple difficult weekends and night-shifts to really get that balance… The hands-on experience, that being put in the moment and being put on the spot and under pressure for me, was the biggest, biggest learning experience.” (Respondent G)*
	Observing more experienced healthcare professionals	12 (6)	*“There are certain things that you will pick up from some medical registrars or medical SHOs in the way that you [think], “I really like how you did that, I will try and incorporate that into my practise”.” (Respondent C)*
	Taking responsibility for decisions	9 (3)	*“You take no responsibility at medical school, you are not forced to make decisions... I did observe people assessing acutely unwell [patients] but was never forced to think myself which is probably the harder thing.” (Respondent E)*
Why reflection matters	Useful learning through reflection with colleagues	3 (3)	*“I think the form of reflection I find the most useful is actually just debriefing or talking to a colleague… I just find that kind of instant feedback that you get with talking it out with someone you trust is really like way more valuable.” (Respondent B)*
	Useful learning through self-reflection	5 (2)	*“I think reflecting albeit just in your own head, not necessarily formally, is essential. Because you cannot change what you have done previously if you don’t reflect.” (Respondent E)*
Experiences of simulation	Simulation doesn’t reflect the uncertainty of real-world	10 (4)	*“[Simulation] doesn’t take into account the uncertainty that you get sometimes. So, that’s why a simulation feels sometimes feels quite an artificial environment.” (Respondent C)*
	Useful for learning to think under pressure	4 (3)	*“[Simulation] actually got you thinking outside of just what’s in front of you and how you treated the team that you are dealing with.” (Respondent F)*
	Minor themes: Useful for learning about MDT More useful as F1 when aware of responsibilities Feedback is on best self rather than true behaviour		*“Yeah, yeah, I think that would be really helpful actually having a nurse and HCA there because then you can get to know what their competencies are... And understand what their respective roles are.” (Respondent C)*
Experiences of undergraduate learning opportunities	Useful shadowing junior doctors and emergency response teams	9 (4)	*“But I think probably the most useful thing would be when you are later on in medical school to be like partnered with an F1 or F2 on-call. And either just observe or where it’s appropriate be like exposed to doing a bit of initial assessment.” (Respondent E)*
	Teaching too theoretical, interested in practicalities of acute care	10 (4)	*“So I think we’re often given the bog-standard cases in medical school, so you’re looking from a textbook, science book, sometimes patients don’t present with the textbook signs.” (Respondent A)*
	Useful when final year aligns with preparing for F1	6 (4)	*“I think that med school can incorporate all of their knowledge within four years... And the fifth year should be something [where] you prepare yourselves for F1.” (Respondent F)*
	Minor themes: No experience of decision making as student Need proactive seniors Lack of continuity in acute care experiences Desire for more MDT learning together		*“...I think the one shame is in medical school and probably in F1, there isn’t more MDT learning together.” (Respondent C)*

“You take no responsibility at medical school, you are not forced to make decisions... I did observe people assessing acutely unwell [patients] but was never forced to think myself which is probably the harder thing.” (Respondent E)

Observing senior clinicians respond and reflecting on how they approached the situation were seen as valuable real-world learning experiences. These experiences were more beneficial if the participant held some responsibility or investment in the case. The opportunity to shadow the medical emergency team was also felt to be a very useful experience.

“Seeing what a registrar does when they come and how they respond to you, the patient, and the situation are all three things that I try to take on board every time.” (Respondent G)

Learning from hands-on experience was directly related to the volume of exposure. Preparedness was felt to develop more quickly following busy on-call or weekend shifts. Participants on busier, more stressful first rotations felt they had become better doctors as a result, and in hindsight felt grateful for these experiences.

“At the time it was bloody miserable… [Once] you got through that period, I felt that I was probably able to make decisions... Which is probably why I’m quite grateful for having that job now.” (Respondent C)

Both self-reflection and informal debriefing with colleagues were seen as essential parts of learning following hands-on experience. Undergraduate simulation was felt to be another essential experience in developing acute care preparedness, mainly as it gave students an opportunity to think and react under pressure. Participants felt simulation after starting work was more useful as they had more awareness of their actual responsibilities as an FY1 doctor. However, a key criticism was that simulation often does not reflect the uncertainty and unpredictability of the real-world.

"[Simulation] doesn’t take into account the uncertainty that you get sometimes. So, that’s why a simulation feels sometimes feels quite an artificial environment." (Respondent C)

In terms of formal teaching during their undergraduate training, participants felt they had limited teaching on acute care, and the teaching they did receive was too theoretical. They felt that formal teaching could be more focussed on the practicalities of acute care, and suggested this may be better delivered by near-peer trainee doctors. There was also a desire for more combined learning experiences with other members of the MDT. Participants felt that more formalised shadowing of junior doctors or medical emergency teams would be very useful experiences.

“But I think probably the most useful thing would be when you are later on in medical school to be like partnered with an F1 or F2 on-call. And either just observe or where it’s appropriate be like exposed to doing a bit of initial assessment.” (Respondent E)

## Discussion

Our results suggest that FY1 doctors may feel unprepared in responding to acutely unwell patients when they start work. This is not a new finding. Their emotional response in these situations has not been studied in-depth previously, and our findings suggest that these early experiences can be challenging and overwhelming. A lack of confidence in what is expected of them and when they should escalate were two major themes related to unpreparedness that we identified in our cohort.

We recognise there will always be a stressful transition from medical student to doctor and, given its nature, acute care will be a common area of concern. As suggested by Tallentire et al. [3], it is probably preferable for patient safety that FY1 doctors never feel completely at ease with acute care and therefore call for senior assistance more readily. Our findings suggest that learning to ask for help is in fact part of how new doctors conceptualise preparedness for acute care and that increasing training, particularly in real-world settings, will not lead to failure to escalate. Challenges on starting work as a doctor, with effective clinical supervision, have also been suggested as important for both personal and professional development [11]. However, given our findings and because preparedness in acute care ranks poorly compared with other outcomes, combined with the risks to patient outcomes, we see this as an important area for improvement in undergraduate medical education.

Participants in our study drew from a range of different learning experiences in developing preparedness. Out of the options presented to participants in the sorting and ranking exercise, experiential learning was consistently ranked top of the list. Similarly, a lack of exposure to acute care at medical school was associated with feelings of unpreparedness. As emergencies are unpredictable, shadowing foundation doctors’ on-call shifts and medical emergency teams are two learning opportunities that could be scheduled into clinical attachments to increase students’ hands-on exposure. As students become core members of this community of practice, effective knowledge translation can occur [12]. Shadowing these teams may also provide an opportunity for a sense of independent practice within a protected environment, allowing students to feel they have some ownership of the situation and experience decision making when appropriate. Our participants also felt that a high volume of exposure was important in developing preparedness, and therefore these learning experiences should be longitudinal rather than seen as a one-off.

The GMC requires final year medical students to undertake a period of shadowing prior to beginning work [13]. Our results suggest this might be too passive to drive students into an effective experiential learning cycle, which appears to be crucial to preparedness. Perhaps this could be reimagined as “reverse shadowing”, where the student takes on fuller responsibilities, shadowed by the outgoing incumbent? This could potentially bring forward this steep learning curve to a time when the supernumerary learner is more adequately supported.

In support of experiential learning theory, reflection was also frequently ranked as an essential part of developing preparedness [14]. Participants mostly described this as informal reflection with colleagues or as self-reflection, and it was particularly useful after difficult experiences. To aid students in reflection, dedicated debrief sessions could be arranged during acute care attachments with supervision and support from experienced medical staff.

Formal undergraduate teaching on acute care could be focussed on basic principles of knowing the hospital environment and MDT, understanding the initial management steps, recognising the seriousness of a situation and knowing when to escalate, targeted specifically at the expectations of an FY1 doctor. It is promising that all participants felt prepared for acute care after three to six months of work experience, and therefore improvements to acute care teaching and workplace experiences as undergraduates could translate to a less overwhelming start for FY1 doctors and improved patient care.

Although simulation was seen as a valuable learning experience, especially for thinking under pressure, our participants expressed concern that simulation does not accurately reflect the timing and uncertainty of a real emergency. The results are similar to that of Tallentire et al. [15] in that simulation often struggles to reflect the stressful and hierarchical environment of clinical practice. Simulation was felt to be more beneficial once participants had started work and better understood their responsibilities as junior doctors. Interestingly, one participant felt that the feedback they received from simulation was on their best self rather than their true behaviour. Simulation is therefore an important tool, but our findings highlight the importance of hands-on learning and effective feedback in the real-world.

Further research could explore other stakeholders’ perspectives of junior doctors’ preparedness in acute care to gain insight into their experiences and expectations. An action research project involving undergraduates “reverse shadowing” junior doctor on-call shifts may provide further evidence of how experiential learning can be effectively facilitated in acute care.

### Strengths and limitations

This study was unique in that participants compared and ranked different educational experiences, which are typically studied in isolation. Junior doctors as participants are a particularly difficult cohort to access due to the high work demands and concerns around discussing unpreparedness safely. A strength of this study was that it was conducted by near-peers in a safe, neutral environment off the ward.

We did not collect data on participants’ undergraduate backgrounds or their first rotations as junior doctors, as their subjective journey to preparedness was our subject of interest. Barnsley et al. [16] previously demonstrated a disconnection between self-reported confidence and formally assessed competence in junior doctors. For this reason, we chose to ask participants if they felt prepared, rather than confident, when responding to deteriorating patients. Given the high stress nature of acute care, it is possible that junior doctors may never describe feeling prepared during their first on-call experiences. Perceived safeness, as suggested by Roland et al. [17], may be useful metric in future research on preparedness as a commonly understood and unifying term for competence and confidence.

As only seven interviews were conducted from a single hospital in London, we cannot say that the full range of experiences or opinions has been captured. It is possible that only junior doctors who felt the most affected or disaffected by their acute care experiences volunteered to be interviewed. Nonetheless, there are strong categories of data that appear consistent across cases. Our study raises important questions about how we prepare medical students for acute care and provides a basis for further research.

## Conclusion

These medical graduates felt unprepared for acute care. From their perspective, preparedness developed over the first three to six months through a range of learning experiences, with hands-on experience, reflection, simulation and MDT team-working ranked the most essential. Although medical schools can never replicate starting work, undergraduate educators should aim to improve medical students’ preparedness in acute care.

Formal undergraduate teaching could be targeted at the expectations of an FY1 doctor with a focus on basic initial management, recognising severity and knowing when to escalate. Our results suggest that the current level of shadowing in acute care may be sub-optimal and simply observing how seniors respond may be too passive. Increasing the intensity of clinical shadowing by putting students into positions where they are learning to make decisions and get feedback on these decisions may improve preparedness and should inform future educational interventions. Preparedness in acute care includes the complex social aspects of learning to work interprofessionally, as well as learning the material aspects of how to navigate the hospital equipment and systems – our findings suggest simulation-based education may fail in these areas if it doesn’t reflect the complexities of real life.

## Data Availability

The datasets used during the study are available from the corresponding author on reasonable request.

## Abbreviations

CQR: Consensual qualitative research
FY1: Foundation year one doctor
GMC: General Medical Council
MDT: Multidisciplinary team
MERU: Medical Education Research Unit
